# Engineering safety into AI-assisted music medicine for headache management: a transparent guardrail framework

**DOI:** 10.3389/fneur.2026.1870266

**Published:** 2026-07-13

**Authors:** Xuexing Luo, Zhichao Pei, Amy Jing-Wen Yin

**Affiliations:** 1 College of Art and Design, Lingnan Normal University, Zhanjiang, Guangdong, China; 2 School of Design, South China University of Technology, Guangzhou, Guangdong, China; 3 Department of Psychiatry, Affiliated Hospital of Guangdong Medical University, Zhanjiang, Guangdong, China

**Keywords:** artificial intelligence, clinical guardrails, digital therapeutics, headache, music medicine

## Abstract

While generative artificial intelligence enables large-scale, individualized, and digitally scalable interventions, its deployment introduces model instability and algorithmic reasoning errors. To ensure patient safety, the next frontier for AI-assisted music medicine therapeutics is not merely tool optimization, but the implementation of a transparent, rule-based clinical safety guardrail.

## Introduction

Headache disorders affect more than 50% of the global population, with migraine and tension-type headache (TTH) constituting the highest burden. Among the over 3 billion individuals affected, the vast majority depend on long-term medication, primarily analgesics such as ibuprofen and acetaminophen. Prolonged use of these drugs frequently leads to drug tolerance and poses serious risks of hepatotoxicity and cardiovascular complications (1, 2). Additionally, socioeconomic and geographical barriers restrict access to specialized neurological care, leaving millions to endure chronic, unmanaged pain (3).

AI-assisted music medicine—the systematic, algorithmically driven administration of acoustic stimuli to achieve targeted physiological or psychological effects—has drawn long-standing attention for its potential pain-suppressing qualities, backed by positive preliminary trials (4, 5). However, a core translation challenge remains: can a uniform music intervention work across profoundly diverse populations? An elderly individual in Shanghai, a teenager in Brisbane, and an office worker in New York vary fundamentally in their musical aesthetics, autonomic nervous system reactivity, and cultural expectations (6). Furthermore, different headache phenotypes involve distinct neural pathophysiologies, meaning an acoustic stimulus that modulates one network could be ineffective or even counter-therapeutic for another (7).

While generative artificial intelligence enables large-scale, individualized, and digitally scalable interventions, its deployment introduces model instability and algorithmic reasoning errors (8). To ensure patient safety, the next frontier for AI-assisted music medicine therapeutics is not merely tool optimization, but the implementation of a transparent, rule-based clinical safety guardrail (9–11).

## Algorithmic music customization

The traditional mismatch between musical stimuli and the listener stems from demographic, clinical, and cultural variance. Factors such as age, ethnicity, biological sex, and cultural background dictate whether a sound configuration induces relaxation or agitation. For example, an older patient may find pain mitigation in a legato melody at 60 beats per minute (BPM) or acoustic white noise, whereas a younger individual accustomed to high-energy genres might find the same tempo monotonous or frustrating, potentially elevating stress and exacerbating head pain (6).

Given the global shortage of specialized healthcare providers, a one-to-one clinical delivery model is unfeasible in most regions, particularly for the continuous monitoring required for real-time personalization (12). Scalable, AI-driven digital interventions provide a viable alternative (13). By processing demographic profiles and real-time physiological feedback, AI models can dynamically align acoustic streams with patient states (Table 1).

**Table 1:** Comparison of human-led and AI-driven music interventions.

Feature	Music medicine	AI-assisted music medicine
Scalability	Can be limited by workforce availability	Global scalability via online app
Neuro-Matching	Manual/iterative matching	High-speed algorithmic mapping
Content Origin	Pre-existing, often with copyright restrictions	Real-time generative
Primary Risk	High cost and limited access for many users	AI hallucination and mismatch
Safety Logic	Clinical expertise and professional judgment	Transparent guardrails and protocols

Furthermore, real-time generative music software lowers production and deployment barriers, enabling patients in economically disadvantaged regions to access high-quality customized audio (14). However, while generative models bypass traditional commercial music licensing bottlenecks, their clinical deployment must navigate complex, jurisdiction-dependent copyright and authorship frameworks governing AI-generated outputs (15).

## Neuro-matching hypotheses and algorithmic vulnerabilities

Primary headaches, including migraine, TTH, and trigeminal autonomic cephalalgias, involve distinct, complex neural circuits spanning the brainstem, hypothalamus, and thalamocortical networks (16). These networks exhibit varying sensitivities to acoustic properties such as rhythm, tempo, and spectral complexity. Theoretically, AI models trained on neuroimaging and electrophysiological data could map a patient’s phenotype to an optimal acoustic configuration more efficiently than human trial-and-error (17). However, the assertion that specific acoustic features can selectively target neural pathways remains a hypothesis that requires rigorous empirical verification.

The primary clinical barrier is not human error, but systemic risks inherent to AI: model hallucinations and processing biases (8). An AI matching program or prompt-to-audio generator may misinterpret patient inputs—particularly among middle-aged or elderly users with limited experience in prompt engineering—resulting in acoustic outputs with inappropriate percussive elements or erratic rhythms. Because generative AI models are probabilistic, their outputs are not deterministic. Without deterministic safety architectures, an AI-assisted music medicine tool intended to relieve pain may inadvertently amplify it (9, 18).

## The transparent clinical safety filter architecture

To mitigate these hazards, we propose that any AI application for headache treatment integrate a deterministic, rule-based clinical safety filter between the generative AI model and the user (19, 20). This safety layer operates transparently using provisional safety parameters, in order to evaluate this methods, we presented 4 provisional design principles:

*P1: Patient Profile & Inputs → P2: Generative AI Model → P3: Transparent Safety Filter → P4: Safe Patient Delivery*.

The operational workflow of the architecture is defined by these provisional pillars:

1. Input Parameters: The system ingests the patient’s verified diagnostic profile (e.g., ICHD-3 classification), demographic baseline, and current pain score.
2. Acoustic Constraints: The provisional safety parameters evaluates the generated audio file against hard clinical limits before it reaches the patient’s headphones. It intercepts any output that violates predefined criteria, such as a maximum allowable tempo (< 100 BPM), sudden transient decibel spikes, high-frequency spectral power concentrations, or syncopated percussive patterns.
3. Human Oversight and Escalation: The platform operates under clinical supervision. If an audio generation cycle fails the safety verifier three consecutive times, the system defaults to a pre-verified, passive acoustic track and logs a notification for the supervising clinician to review the model’s performance.
4. Target Population and Selection Criteria: The trial will recruit adult patients (ages 18–65) meeting the International Classification of Headache Disorders (ICHD-3) criteria for either episodic migraine or tension-type headache (TTH), who experience between 4 and 14 headache days per month. Primary exclusion criteria include secondary headache disorders, severe cognitive or communicative impairments, structural hearing loss, or recent alterations to prophylactic headache medications within the past 30 days.
5. Randomization and Stratification: Participants will be randomized in a uniform 1:1:1:1 allocation ratio across the four evaluation arms. To control for underlying pathophysiological variations and baseline clinical heterogeneity, randomization will be executed via a computer-generated block randomization sequence and explicitly stratified by primary headache subtype (Episodic Migraine vs. Tension-Type Headache).
6. Sample Size Estimation: Assuming a projected medium Cohen’s effect size (*f* = 0.25) for the primary efficacy endpoint, a statistical power of 80% (1 − beta = 0.80), and a two-sided significance threshold of alpha = 0.05, a minimum sample size of 152 evaluable participants is required across the four parallel arms. To adequately protect the trial against an anticipated 15% drop-out or attrition rate over the course of the longitudinal evaluation, the total target recruitment size is set at *N* = 180 participants (45 participants per arm).
7. Adverse-Event Monitoring and Safety Escalation: Continuous safety monitoring will be mediated through a digital symptom-tracking diary integrated directly within the application. Participants will be mandated to log immediate post-session responses. The primary safety endpoint is operationally defined as “music-induced headache exacerbation,” characterized by an immediate increase in pain intensity of > = 2 points on a standard Visual Analog Scale (VAS) within 30 min of starting the audio session, or the acute onset of severe phonophobia, photophobia, or nausea. Any such event will trigger an automated safety protocol: immediate termination of the generative audio feed, automatic default to a pre-verified passive baseline track, and an immediate electronic alert escalated to the principal investigator within 24 h.
8. Statistical Analysis Plan (SAP): All primary and secondary efficacy evaluations will be conducted utilizing an Intention-to-Treat (ITT) analytical approach. The primary outcome—the change in mean monthly headache frequency from baseline to the 12-week post-intervention milestone—will be analyzed using a Linear Mixed-Effects Model for Repeated Measures (MMRM). This model will treat time, treatment arm, and the time-by-treatment interaction as fixed factors, while incorporating baseline headache frequency and the stratified headache subtype (migraine vs. TTH) as baseline covariates. Missing data will be handled assuming a missing-at-random (MAR) mechanism embedded within the mixed-effects framework. Pairwise post-hoc comparisons between arms will be adjusted for multiplicity using the Bonferroni correction.
9. Multi-center, four-arm parallel RCT: *Arm A* (Standardized Passive Control): Patients listen to pre-recorded, static relaxation music. *Arm B* (Unverified AI-Matched Music): Patients receive dynamically generated music customized by an AI model without an intermediary safety filter. *Arm C* (Verified AI-Matched Music): Patients receive dynamically generated music passed through the transparent clinical safety filter. *Arm D* (Usual-Care Control): Patients continue their standard pharmacological and non-pharmacological routines without an audio intervention.
10. Primary Outcomes: Change in mean monthly headache frequency and pain intensity (measured via a Visual Analog Scale) from baseline to a 12-week follow-up.
11. Secondary Outcomes: Acute medication intake frequency, quality of life scores (HIT-6), and adverse event rates.

## Conclusion

Precision AI-assisted music medicine holds substantial promise for accommodating individual preferences, addressing neuroanatomical variables, and democratizing access to non-pharmacological interventions. However, without a dedicated clinical safety architecture, digital therapeutic deployment remains high-risk. Ultimate clinical and legal responsibility rests with human clinicians and developers; the digital devices deployed into patients’ homes must be architected with deterministic safety mechanisms to ensure they protect patient well-being under all conditions.

## Data Availability

The original contributions presented in the study are included in the article/supplementary material, further inquiries can be directed to the corresponding author.
